# Visual Recognition of Traffic Signs in Natural Scenes Based on Improved RetinaNet

**DOI:** 10.3390/e24010112

**Published:** 2022-01-12

**Authors:** Shangwang Liu, Tongbo Cai, Xiufang Tang, Yangyang Zhang, Changgeng Wang

**Affiliations:** 1 School of Computer and Information Engineering, Henan Normal University, Xinxiang 453007, China; caitongbo@outlook.com (T.C.); tang15103739221@163.com (X.T.); zhangyangyang@caas.cn (Y.Z.); wcg1697997028@163.com (C.W.); 2 Henan Engineering Laboratory of ‘Smart Business and Internet of Things Technology’, Xinxiang 453007, China

**Keywords:** RetinaNet, natural scenes, traffic signs, ResNeXt, group normalization

## Abstract

Aiming at recognizing small proportion, blurred and complex traffic sign in natural scenes, a traffic sign detection method based on RetinaNet-NeXt is proposed. First, to ensure the quality of dataset, the data were cleaned and enhanced to denoise. Secondly, a novel backbone network ResNeXt was employed to improve the detection accuracy and effection of RetinaNet. Finally, transfer learning and group normalization were adopted to accelerate our network training. Experimental results show that the *precision*, *recall* and *mAP* of our method, compared with the original RetinaNet, are improved by 9.08%, 9.09% and 7.32%, respectively. Our method can be effectively applied to traffic sign detection.

## 1. Introduction

Traffic sign recognition is an important technology for environment perception in autonomous driving and high-definition map (HD Map), which can offer road information judgments for safe vehicle driving and provide real-time security warning, ensuring driver safety. However, the result of traffic sign recognition is still limited by many factors due to diverse road conditions and the natural environment [1]. When the illumination changes, the occluder is covered, and the sign information is blurred, it is difficult to detect and recognize target signs.

For the object detection task, the popular detection frameworks are Faster RCNN [2], YOLO [3], and SSD [4], etc. YOLO and SSD are one-stage object detection algorithms that may directly deliver object category and location information via regression, which is considerably faster than the two-stage technique. However, its accuracy is poor and the object recognition result may not achieve the optimal state. Faster RCNN is of two-stage technique that has high accuracy but slow speed since it first utilizes regression to create a series of anchors and then uses convolutional neural networks to categorize them. Facebook AI research team produced good results by upgrading the loss function and therefore proposed the RetinaNet [5], a detection framework to establish a combination between accuracy and speed in detection tasks. Since the standard RetinaNet uses ResNet [6], the current ResNeXt [7] network has fewer hyperparameters and simpler structure than ResNet by using group convolution in the network layer with the same guaranteed parameters. So, it could assist increase the RetinaNet’s target detection performance, and decrease the false and missed rate of tiny targets in challenging situations, and improve the model’s robustness and reliability, as well as improving traffic sign detection. Therefore, we propose the RetinaNet-NeXt framework, a detection framework for detecting traffic signs in natural scenes. Our method recognizes traffic signs by using ResNeXt to extract bottom-up features from the input image, a laterally connected FPN to fuse top-down, low-level, high-resolution info and high-level features with rich semantic information, and a convolutional network to classify and predict. A lot of experiments are carried out on the basis of Tsinghua-Tencent 100K [8] traffic sign dataset to verify the efficiency of the proposed approach. The proposed method can effectively recognize traffic signs in natural scenes and achieves better accuracy than the current RetinaNet with a higher *mAP*.

This paper is organized as follows: Section 2 reviews previous work on traffic sign recognition. Section 3 presents our method. Section 4 presents the experiments and results, and Section 5 is dedicated to the conclusions.

## 2. Related Work

Many researchers have attempted to increase the accuracy of traffic sign recognition using a variety of methods [9,10,11,12,13,14,15,16,17,18,19,20,21]. In general, there are three categories of traffic signs in China: indication, warning and prohibition, which are represented by blue, yellow and red, respectively. It has a variety of shapes, such as circle, triangle, and rectangle. Color and shape features are now used to detect traffic signs, with K-mean method used for color grouping and a convolutional neural network used for detection [22]. Dai Xuerui et al. [23] used different color thresholds, maximally stable extremal regions (MSER) to detect traffic sign, combined with shape features to judge after discovering the region of interest, and used support vector machine (SVM) and histogram of oriented gradient (HOG) features to classify the area, but for different traffic signs, the threshold value must be re-adjusted. Xin Wu et al. [24] fused the channels of traffic sign color information to achieve contrast enhancement and background noise reduction, improving recognition accuracy and robustness, but they fail to detect traffic signs with similar shapes and colors.

With the development of deep learning, Yanmei Jin et al. [25] improved the detection of small and medium-sized targets through multi-feature fusion and enhancement by enhancing effective channel features and suppressing ineffective channel features to enhance features, which are less effective in recognizing small targets and cannot achieve high accuracy due to learning through lower-level features; Xu Jindong et al. [26] proposed a reliability-based spatial context fuzzy c-means algorithm (RSFCM) for suppressing pretzel and Gaussian noise in image segmentation based on spatial contextual information, improving the performance of traffic sign images on the clustering algorithm, and achieving effective retention of image edge information. Prakash et al. [27] used Gabor filter and Adam optimizer for feature extraction of LeNet to improve the processing capabilities of the model in image recognition and achieved high classification results on GTSRB [28] dataset, overcoming the drawbacks of traditional traffic sign recognition methods such as higher computing complexity and lower detection accuracy. To improve the robustness of traffic sign recognition, Haobo Lv et al. [29] utilized Yolov3, incorporated noise data, and used soft NMS. Huan Zhu et al. [30] adopted Focal Loss to solve the problem of difficult distinction between positive and negative samples in the GTSDB [31] dataset of German traffic signs, and achieved 88.23% mAP. Zhilong He et al. [32] built a lightweight convolutional neural network TS-CNN with a 10-layer architecture using simple convolution and pooling operations, with a detection accuracy of 99.3%, but they lacked attention and research on image recognition in natural scenes and complex weather conditions. Qiuyu Zhang et al. [33] changed the network structure of VGG to six layers [34], introduced high-level features and capture the contextual information of feature map by block-cross, improving the real-time and accuracy of detection of smaller traffic signs. Zha, M. et al. [35] used feature pyramid network and coordinate attention [36] for forestry pest detection, and obtained 38.62% mAP on the COCO dataset based on MobileNetv2. Yang, L et al. [37] proposed a vehicle multi-feature detection algorithm based on binocular cameras, combined with feature pyramid network to realize the recognition of the three characteristics of license plate, sign and light, improving the robustness of vehicle speed measurement.

## 3. The Proposed Methods

**Figure 1:**
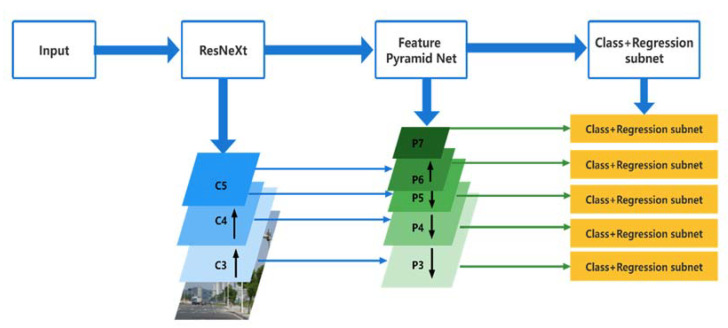
Structure of RetinaNet-NeXt network.

As shown in Figure 1, in this study, the feature extraction network (ResNet) is replaced with ResNeXt, and the feature pyramid network (FPN) is adopted to overcome the multi-scale problem in object detection, and Group Normalization (GN) [38] is employed to maintain training accuracy, and the loss function is Focal Loss.

Figure 1 shows how the backbone network extracts bottom-up features from the input image using ResNeXt. ResNeXt is divided into several subgroups for convolution, in contrast to the common ResNet feature extraction network in the RetinaNet, and the data for each channel is computed independently while performing the convolution. As the number of hyperparameters is reduced, the validation error is reduced while more subspaces are used. For the output of con3v, conv4 and conv5 layers, the residual block output of ResNeXt is represented by {C3, C4, C5}. Then, five feature layers are extracted from the network to construct the feature pyramid network FPN, and the feature layers are represented by {P3, P4, P5, P6, P7}. Finally, two different Fully Convolution Network (FCN) classification subnet and regression subnet, are connected as network outputs. The classification subnet classifies the output and acquires the class label of the object, while the regression subnet uses convolutional bounding box regression to determine the position.

### 3.1. Feature Extraction Network

**Figure 2:**
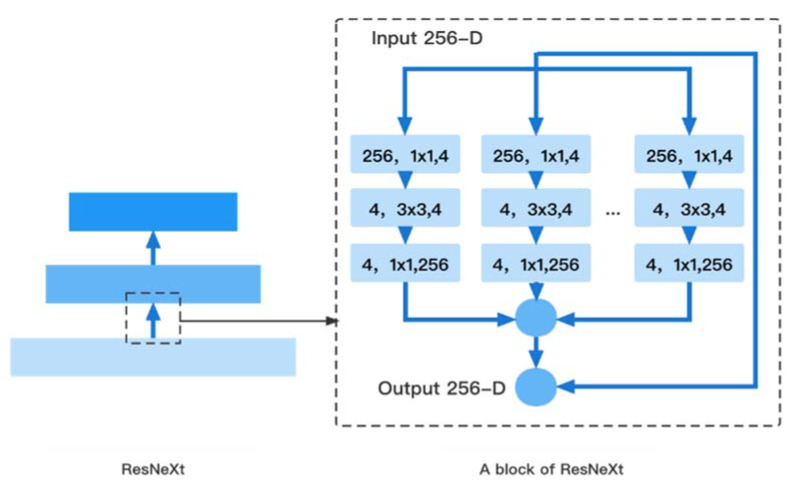
Backbone Network (ResNeXt).

To achieve more effective detection, ResNeXt is regarded as the backbone network for feature extraction of the input image. ResNeXt is built on ResNet’s modular structure and incorporates the high recognition performance of split-transform-merge in Inception. The right side of Figure 2 shows the structure of each basic unit.

In Figure 2, ResNeXt uses multiple convolution modules to perform feature extraction from bottom-up, and group convolution uses the same topology on different input channel paths. By using cardinality as a super parameter, it’s able to achieve a more efficient network. For a 256-dimensional input with cardinality of 32, the network encodes 256 channels into 4 channels, and the features are extracted in 32 different embedding spaces by 32 different groups consisting of continuous 1 × 1 conv, 3 × 3 conv, and 1 × 1 conv.

### 3.2. Feature Pyramid Network

**Figure 3:**
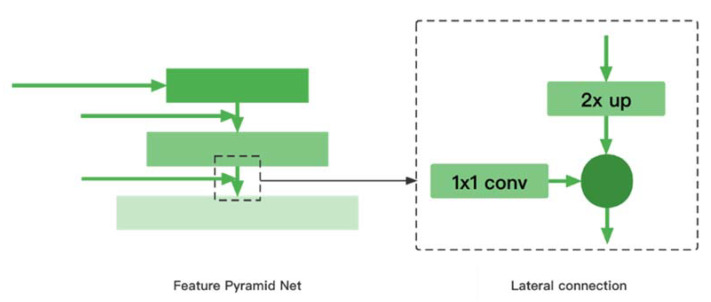
Structure of Feature Pyramid Network.

After the feature is extracted by using ResNeXt, the network laterally connects the top-down feature pyramid network (FPN) to fuse with the bottom-up ResNeXt feature layer. The FPN structure is shown in Figure 3.

In Figure 3, each basic unit in the feature pyramid network is fused with features from the higher levels of upsampling by laterally connecting top-down feature extraction layers of ResNeXt that have the same spatial size. The P3, P4 and P5 layers in the FPN are obtained by laterally connecting the C3, C4 and C5 layers from feature extraction. P6 is obtained by convolving C5 with a 3 × 3 conv kernel and a step size of 2. P7 is obtained by applying the Relu function to P6 of operation with a convolution kernel of 3 × 3 and a step size of 2. Since the feature map from higher layers has a smaller size, it is more conducive to feature expression and facilitates the detection of larger objects. the feature maps from lower levels, P3, P4 and P5, are more suitable for the detection of small objects due to their high resolution. Through the feature pyramid network, the model has stronger ability and better result in feature expression, while the amount of computation for object detection remains the same.

### 3.3. Classification and Regression Subnets

**Figure 4:**
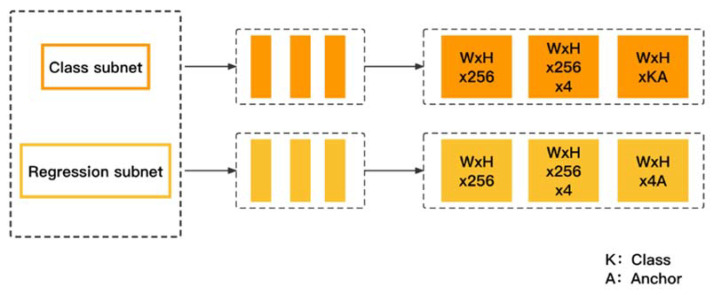
Structure of classification and regression subnet.

The classification subnet and regression subnet are fully convolutional networks (FCN) connected to each FPN level. Classification subnet is used to predict the probability of each anchor and K-class object appearing in each spatial position. Similarly, the regression subnet is used to regress the offset of the bounding box, but the parameters are not shared. If positive samples can be detected, the object position relative to the anchor will be output. Figure 4 shows the classification and regression network structure.

In Figure 4, the classification subnet consists of four 3 × 3 conv layers with 256 filters, each of which is activated by the Relu function. The other 3 × 3 conv layers consist of K × A filters, each activation using a sigmoid function. The classification subnet has shared parameters across all levels, the shape of the output feature map is (W, H, K, A), and the size is W × H × K × A. W and H are proportional to the width and height of the input feature map, and the object class and anchor numbers are denoted by K and A, respectively. The design of the regression subnet is similar to that of the classified subnet, except that the number of output channels is 4A. It adjusts the bounding box by calculating the offset between the ground truth and the anchor, and the final conv layer is a 3 × 3 conv composed of 4 filters. Therefore, the output feature map has the shape (W, H, 4, A) and a size of W × H × 4 × A.

### 3.4. Group Normalization

The images are input to the training network with the image shape [N, C, H, W] in the network, where N represents the batch size, C denotes the number of channels, H indicates the image height, and W means the image width. Affected by the network characteristics of RetinaNet, Batch Normalization (BN) [39] depends on the batchsize, for general GPUs, the batch size can often only be very small values such as 2, 4 and 8, and the error calculated by a smaller batch size is larger, and the model’s error rate is easier to rise, and the training network is worse at object detection [40]. To solve the problem, we adopt Group Normalization (GN) in network training, replacing BN in the standard ResNeXt. The group normalization operation is implemented by calculating the mean and variance of each group, which divides the channels into 32 groups to speed up the training of the whole network while reducing the error loss, avoiding the effect of batch size on the model. The method for calculating group normalization is shown in (1).

(1) $$y=\frac{x-E(x)}{\sqrt{Var(x)+}\varepsilon }\times \gamma +\beta ,$$

here *x* is the feature computed by a layer; *E*(*x*) is the mean; *Var*(*x*) is the variance; γ and β are scaling and panning factors, respectively; γ takes values in the range of (0, 1), and β is set to 0 by default. Setting affine = True activates weight (γ) and bias (β), which can be panned and scaled when calculating the normalized results in the specific implementation.

### 3.5. Focal Loss

Focal Loss is a cross-entropy loss function that reduces the proportion of negative samples in the sample data during the training process to address the loss of network accuracy caused by the imbalance of positive and negative samples in the target detection task. The cross entropy could be calculate from Equation (2).

(2) $$CE(p,y)=\left\{ \begin{array}{l} -\log(p),\;\mathrm{if}\;y=1\\ -\log(1-p),\;\mathrm{else} \end{array}\right.,$$

where *p* is the probability that the predicted model belongs to the class *y* = 1.

Thus, *p* is calculated as follows:

(3) $$p_{t}=\left\{ \begin{array}{l} p,\;\mathrm{if}\;y=1\\ 1-p,\;\mathrm{else} \end{array}\right.,$$

There may be a large number of iterative processes of simple samples in Equations (2) and (3) that cannot be optimized to the optimum, which has a significant impact on the detector. Hence, the first step in improving Focal Loss is to add γ to the original basis to reduce the loss of easy samples and balance the hard and easy samples, as shown in (4).

(4) $$L_{\mathrm{fl}}=\left\{ \begin{array}{l} -{\left(1-y^{'}\right)}^{\gamma }\log y^{'},\;y=1\\ -y^{'\gamma }\log(1-y^{'}),\;y=0 \end{array}\right.,$$

Meanwhile, a coefficient factor α is introduced to solve the problem of positive and negative sample imbalance, please see Equation (5).

(5) $$L_{\mathrm{fl}}=\left\{ \begin{array}{l} -\alpha {\left(1-y^{'}\right)}^{\gamma }\log y^{'},\;y=1\\ -(1-\alpha )y^{'\gamma }\log(1-y^{'}),\;y=0 \end{array}\right.,$$

Focal Loss can reduce the weights of a large number of simple negative samples by adding a coefficient factor to the cross-entropy calculation, and thus effectively improve the accuracy of the model.

## 4. Experiments and Results

On Tsinghua-Tencent 100K dataset, Experiments are conducted to verify the effectiveness of the improved RetinaNet method. The setup of the experiment is as follows. Software environment: operating system, Ubuntu 20.04; programming language, Python 3.7; deep learning framework, PyTorch 1.8. Hardware environment: CPU, Intel i7-4790 (Intel, Mountain View, CA, USA); GPU, NVIDIA GTX TITAN X (video memory, 12 G) (NVIDIA, Santa Clara, CA, USA); RAM, 32 G.

### 4.1. Dataset and Augmentation Methods

**Figure 5:**
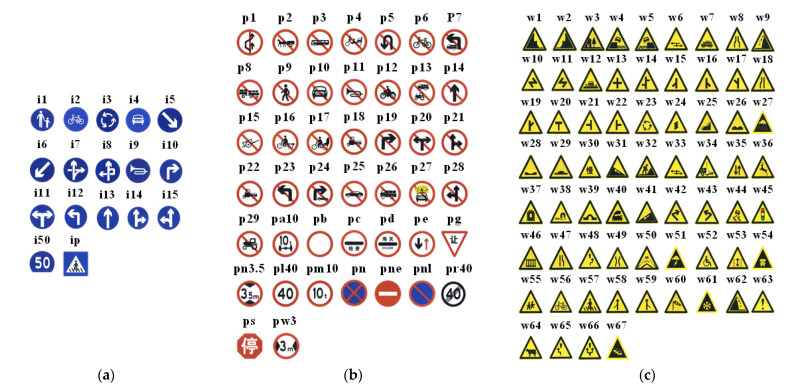
The types of traffic signs in the public dataset TT100K. (**a**) instruction; (**b**) prohibition; (**c**) warning. It is mainly used in this work.

Tsinghua_Tencent_100K is a traffic sign dataset made public by Tsinghua University and Tencent in 2016. Tsinghua_Tencent_100K dataset, in contrast with the GTSDB for detection task and the GTSRB dataset for classification task proposed in Germany, more closely resembles natural scenes and the effects of real-life traffic sign views. The dataset contains about 100,000 panoramic images of natural scenes in various lighting and environments, with about 10,000 images contain 30,000 traffic signs. The dataset contains a total of 3 major categories of sign information, which are instruction signs, prohibition signs and warning signs. Figure 5 illustrates the types of images for this study, and the number of images in dataset is shown in Table 1.

As shown in Table 1, the total number of images for training is 7196, and the number of images for testing is 3071.

Please note that, for some cases in the dataset where the number of samples is too small, the traffic sign classes that take less than 100 occurrences are omitted. Finally, there are 42 sub-classes in our dataset.

**Figure 6:**
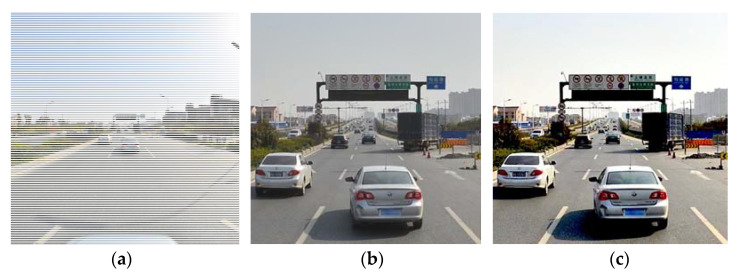
Data augmentation results of traffic sign image: (**a**) Original image; (**b**) Size cropping; (**c**) Color change.

We also apply data augmentation strategies into expanding the dataset via size cropping and color changes in order to improve the model’s prediction performance. After data augmentation, the final training dataset contains 37,212 images. Figure 6 depicts some classical image augmentation results.

In Figure 6, the original image is scaled and cropped to 512 × 512 pixels at random. Following the crop, the illumination interface of TensorLayer’s tl.prepro toolbox is employed to process the image’s lighting information, primarily involving image brightness, saturation, and contrast changes, while taking into account the effects of different lighting conditions on the image such as dimness and blur. The brightness, saturation, and contrast optimization parameters for illumination were set to (0.5, 1.5), and a random combination of variations was utilized to generate multiple resultant image data.

### 4.2. Evaluation Metrics

*Precision*, *recall*, *PR* curve (*precision*-*recall*, *PR*), *F*1-Score, average precision (*AP*), and mean average precision (*mAP*) are adopted as evaluation metrics of the results for model evaluation in order to evaluate the effectiveness of the proposed method for traffic sign recognition. The precision indicates how many of the model’s detected objects are target, and the recall indicates how many of all true targets are detected by the model, which are calculated from Equations (6) and (7).

(6) $$precision=\frac{TP}{\left(TP+FP\right)},$$

(7) $$recall=\frac{TP}{\left(TP+FN\right)},$$

where, *TP* denotes the number of correctly predicted items; *FP* means the number of incorrectly identified items; and *FN* denotes the number of identified items.

As shown in Equation (8), the *F*1 score is an evaluation metric that measures the accuracy of a classification model by calculating the average of precision and recall.

(8) $$F_{1}=2\times \frac{precision_{}\times recall_{}}{precision_{}+recall_{}},$$

By averaging the precision, *AP* is used to assess the model’s strengths and weaknesses in each class. The area enclosed by precision and recall is known as the PR curve. As a result, the calculation is carried out using integration, as shown in Equation (9).

(9) $$AP(n)=\int_{0}^{1}p(r_{n})dr_{n},$$

where *n* denotes a class, $r_{n}$ means the recall belonging to a class *n*, and $p(r_{n})$ denotes the precision corresponding to a class n in the *PR* curve.

*mAP* is the average of *AP* classes, which is used to show the model’s advantages and disadvantages across all classes A the calculation is as follows:

(10) $$mAP=\frac{1}{N}\sum_{n=1}^{N}AP(n),$$

where, *N* represents all classes.

### 4.3. Effectiveness Experiments

**Figure 7:**
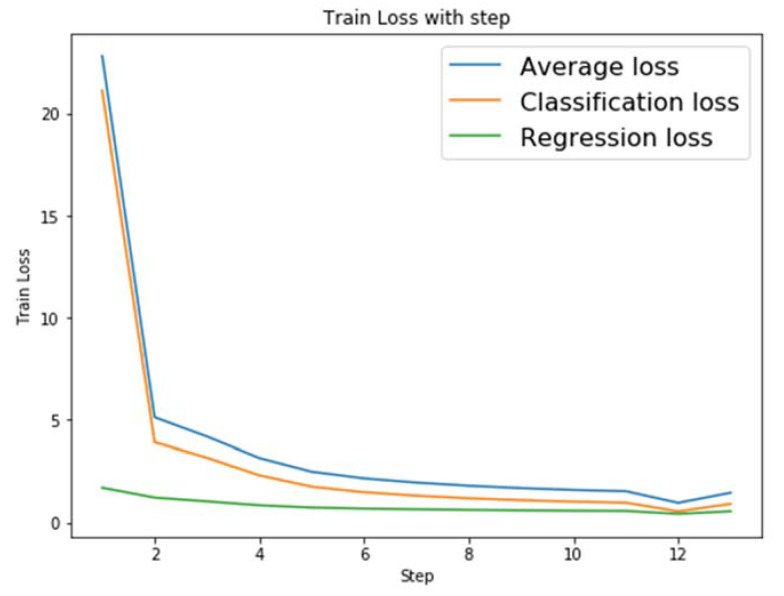
Training loss curve.

We take RetinaNet for detection framework, ResNeXt for the feature extraction network, group normalization for the normalization strategy, ResNeXt-50 for the pre-training model based on ImageNet, and one NVIDIA GTX TITAN X GPU for training. Since the image size in Tsinghua-Tecent 100K dataset is 2048 × 2048 pixels, which is not conducive to training, so it is resized to 512 × 512 pixels. The size of the anchor is set to (0, 512] due to the presence of many smaller traffic signs in the images, and aspect ratios are 0.8, 1, and 1.15 for adjusting the aspect ratio. Scale_ratios are 0.7, 1, and 1.42 for adjusting the area size of the anchor. The batch size is 8; momentum is 0.9; weight decay is 2 × 10^−4^, and learning rate is 1 × 10^−5^ during the training process. Figure 7 shows the variation in network training loss.

The average loss, classification loss, and regression loss of the training model are shown in Figure 7. The cross-entropy loss function is used for classification, while the smooth L1 loss function is used for regression. After several iterations of training processing with ResNeXt and group normalization strategy, the training network’s classification and regression loss decline curves flatten out and the model tends to converge.

**Figure 8:**
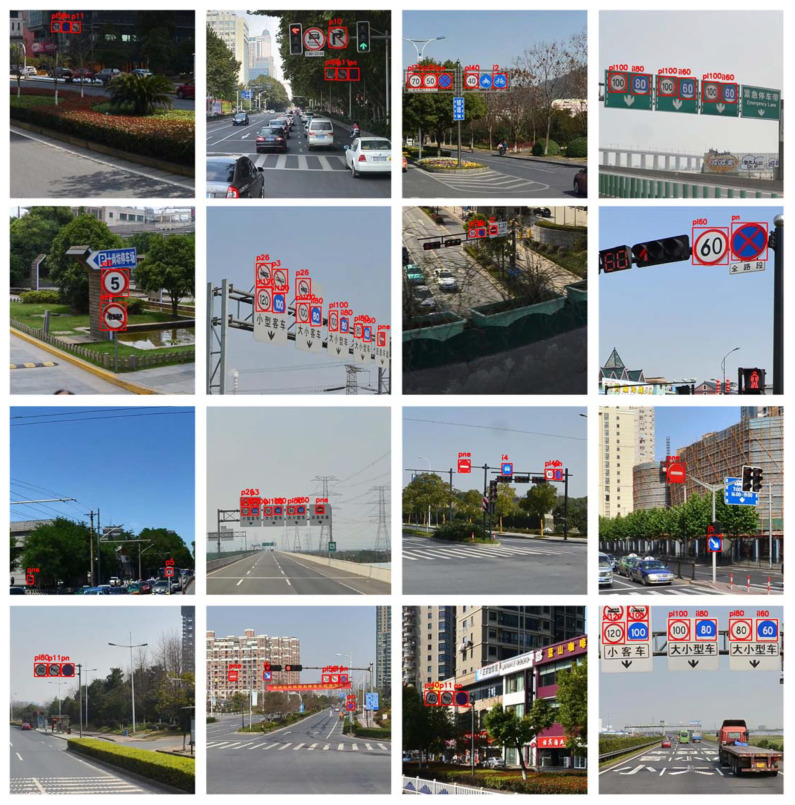
Traffic sign recognition results.

Some classical traffic sign recognition results of our method are demonstrated in Figure 8.

As shown in Figure 8, our method correctly detects a majority of the targets. The recognized signs are marked by red rectangular boxes highlighting the sign areas in the image and predict the sign classes, which include a variety of sign types such as instructions, warnings, and prohibitions. Various roads, neighborhoods, and highways are featured in the test scenes from various perspectives. Signs vary in size and visibility depending on observation distance. As a result, a more detailed anchor division can maintain sensitivity to small targets, allowing the network to focus on them during feature extraction. At the same time, detection is difficult under the influence of factors such as illumination and target visibility, especially when the target object scale is small. The adopted data preprocessing and balancing strategies can decrease the sample detection error caused by external environment changes, which improves the classification effect greatly. In a word, our target detection method is effective in recognizing signs with multi-scales, observation angles, and color changes.

The accuracy of traffic sign recognition under various anchors is given using the ResNeXt model. All detection frames with an IOU < 0.5 are discarded when IOU = 0.5 is used as the threshold, and the accuracy and recall of different anchors are given in Table 2.

The precision and recall for various anchor sizes are shown in Table 2. To improve the ability to predict multiple classes while adjusting the threshold, each anchor has a K-dimensional vector and a 4-dimensional regression vector. The ground truth is matched through the anchor in the K-dimensional label, the exact label is set to 1, and the other is set to 0. When the IOU of an anchor with ground truth is greater than 0.5, it is recorded as a positive sample, and when the IOU is [0, 0.4], the anchor will be ignored in the training process. When the anchor size is (32, 96], the algorithm achieves the highest precision of 90.79% for traffic sign recognition and also the highest value of 86.22% for recall, indicating that the algorithm is the best for the recognition of anchors of size (32, 96], and the size of the detected target object with aspect ratio is closer to the size of the anchor. For smaller or larger anchors, only one anchor can be assigned per object when the IOU of all the anchor’s objects is less than 0.5. When the offset is large, the final regression is inaccurate, the number of positive samples is insufficient, and the detection is worse, resulting in a reduction in the precision and recall of the model.

**Figure 9:**
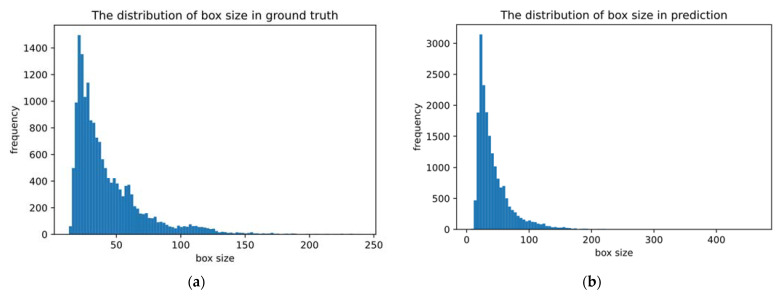
Distribution of ground truth and prediction: (**a**) The distribution of box size in ground truth; (**b**) The distribution of box size in prediction.

In addition, to further explore the effectiveness of the training model intuitively, the ground truth of the dataset and the prediction distribution of the model were analyzed, as shown in Figure 9.

As shown in Figure 9, the ground truth and prediction have similar distributions. Anchor is primarily distributed in the range of (0, 200), with the largest number in the range of (0, 100), confirming the previous discussion that anchor is more evenly distributed between (0, 32] and (32, 96]. Simultaneously, the distribution results show that small target objects account for a larger proportion of traffic signs, and whether a suitable processing method can be adopted for them will have direct impact on the model’s performance and the final results, so small target detection recognition should be paid attention to and optimized.

### 4.4. Comparison and Analysis of Different Detection Frameworks

As well as the RetinaNet, Faster RCNN and YOLOv5 are chosen for comparison experiments based on the TT100K dataset. Faster RCNN’s training setup is as follows: Network is ResNet101, and epoch is 20. YOLOv5 training configuration is as follows: CSPdarknet is network, and Adam is chosen as the optimizer, and the learning rate is 1 × 10^−4^, weight decay is 1 × 10^−5^, and the epoch is 30. Table 3 shows the results of these related method.

Compared with the related methods, the proposed RetinaNet-NeXt achieves better results in *precision*, *recall*, *F*1-Score and *mAP*, reaching 87.45%, 79.65%, 83.37% and 86.71%, respectively, which is higher than the accuracy of standard RetinaNet, YOLOv5 and Faster RCNN in traffic sign detection and recognition.

**Figure 10:**
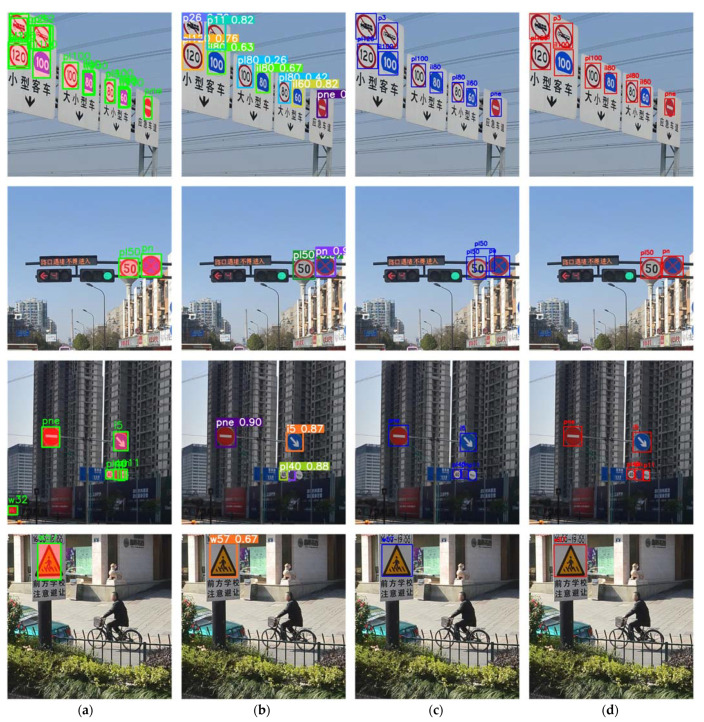
Comparison of recognition results of different detection frameworks: (**a**) Faster RCNN; (**b**) YOLOv5; (**c**) RetinaNet; (**d**) RetinaNet-NeXt.

Furthermore, the recognition result of traffic signs in natural scenes using the above detection framework is shown in Figure 10.

From Figure 10, we can see that, compared with YOLOv5, RetinaNet is able to solve these problems of omission, fall-out and poor object recognition accuracy, and even achieves the same results as the Faster RCNN, two-stage detection algorithm in the recognition of traffic signs in natural scenes, more importantly, we further improve the network model based on the standard RetinaNet. Subsequently, the group convolution strategy and group normalization ensure our network parameters and training loss are as few as possible. Therefore, the extraction and learning ability of our network for target features extraction is strong enough, and our network’s generalization ability is strong too, thus improving the traffic sign detection algorithm’s recognition result under diverse natural scene conditions to the greatest extend.

### 4.5. Comparison and Analysis of Different Models

Furthermore, the performance of various ResNet50, ResNet101 and ResNet152 models with ResNeXt was evaluated by using the RetinaNet based on the TT100K dataset to compare the upgraded network models for the object detection task. Table 4 shows the *precision*, *recall* and *mAP* of these models.

From Table 4, it can be concluded that the feature extraction network was upgraded from ResNet50 to ResNeXt50 based on the original RetinaNet, and the model precision, recall and mAP reached 87.45%, 79.65% and 86.71%, respectively, while keeping the parameters, and the recognition accuracy was improved by 9.08%, 9.09% and 7.32%. Meanwhile, we also tested the performance of RetinaNet under deeper networks such as ResNet101 and ResNet152. The accuracy of the ResNeXt50 model was nearly close to the performance of both, with only difference of 2.55% and 3.09%. Although there is somewhat difference of about 9% in recall, and about 6% in mAP. Compared with the ResNet residual block, ResNeXt changes the original 3 × 3 convolution to group convolution, reducing the input channels overall. The original 128-channel convolution becomes a group of 32 different convolution kernels, and the input and output channels of each convolution are changed from 128 to 4 (128/32 = 4). The total number of output channels remaining the same as the final output will be operated by connection, reducing the network parameters and changing the intermediate channels from 64 to 128 to achieve an increase in network width. With the same parameters, ResNeXt achieves better results than ResNet. That is to say, our group strategy is effective. As the training model converges, the ResNeXt50 model is able to approximate the performance of ResNet101 and ResNet152 at a much faster rate. For large-scale data and model training, the group convolution feature of ResNeXt allows it to effectively reduce the occurrence of overfitting in the dataset and obtain good network performance while keeping the number of parameters constant. All of this results in that the processing and generalization ability of our method can be improved.

### 4.6. PR Curves and Analysis of Different Models under the Effect of Anchor

Combining different network models and anchor sizes, ResNet50, ResNet101, ResNet152, and ResNeXt50 were used to study the impact of anchor size on the accuracy of the model in the range of (0, 32], (32, 96], (96, 512], and (0, 512], respectively, as shown in Table 5.

In Table 5, only using ResNeXt50 as the backbone network of RetinaNet has a greater improvement in the recognition result than ResNet50. For small targets with anchors in the range of (0, 32] and (32, 96], the precision and recall are Compared with the latter, the rate increased by an average of 9.8% and 10.0%. For large targets in the range of (96, 512], the network model is able to achieve the detection precision of 90.29%, which exceeds the 88.94% precision value of ResNet50 and is close to the 90.49% recognition precision of ResNet101 and 90.58% of ResNet152, but the recall is relatively low. This is because precision and recall have a restricted connection, especially for large-scale data, and both of them need to be used optimally for the target task according to the actual situation, which is why the goodness of the target task is always evaluated on various scales using multiple metrics.

**Figure 11:**
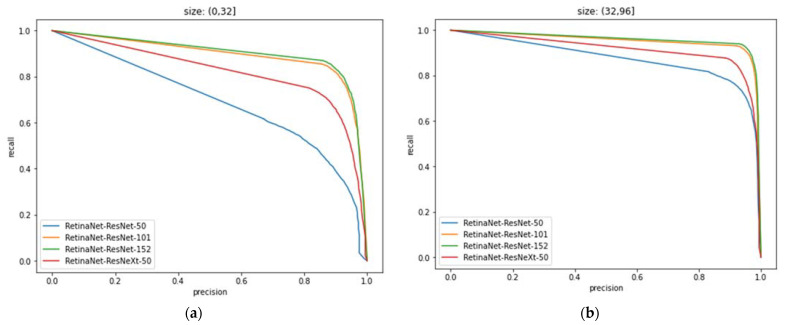

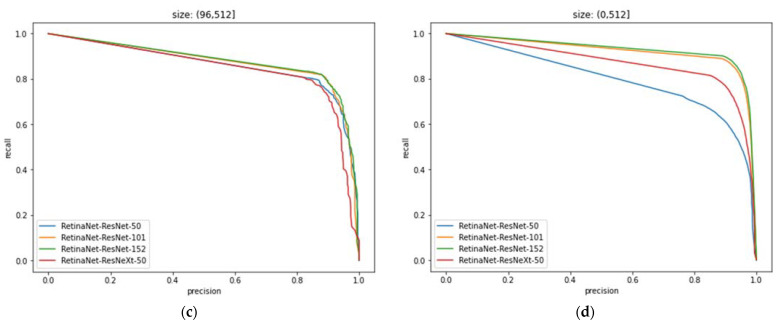
PR curves of different models under the effect of anchor: (**a**) The anchor size is (0, 32]; (**b**) The anchor size is (32, 96]; (**c**) The anchor size is (96, 512]; (**d**) The anchor size is (0, 512].

To further evaluate the network model, the PR curve corresponding to different models is given in conjunction with the anchor, as illustrated in Figure 11.

In Figure 11, the PR curve can clearly reflect the effect of anchor on the model, and then can determine whether the dataset contains hard and wrong samples. Since different anchor sizes correspond to different sample sizes, the data can be adjusted for different or unbalanced training samples to optimize the model performance.

## 5. Conclusions

We proposed a RetinaNet-NeXt method for traffic sign detection in this paper. To reduce data noise and improve the model’s robustness, the dataset’s images are cropped and the data is augmented, and all of the training parameters and anchor sizes are carefully adjusted. Then, the backbone network was replaced with ResNeXt, and the batch normalization was improved to group normalization in the network, eliminating the computational bias caused by small batch normalization effectively. Finally, the validation set’s test results are given as well as the model’s precision, recall, and mAP. The experimental results show that our method can effectively detect traffic sign, especially has a considerable improvement in small target detection while reducing the computational cost. When only using the ResNeXt50 model, our algorithm achieves 87.45% and 79.65% recognition *precision* and *recall*, respectively, and 86.71% for *mAP*. However, there are still many cases of small number of traffic signs and small target objects need to be collected, and how to increase recognition accuracy from limited data, improve the efficiency of network training, and how to implement the lightweight of our model, and how to support the intelligent transportation are the topics that deserve us to study in future.

## Figures and Tables

**Table 1:** The number of images in TT100k dataset.

Type	Size	Total
Train	2048 × 2048	7196
Test		3071

**Table 2:** Precision and recall of different anchor.

IOU	Size	Precision (%)	Recall (%)
0.5	(0, 32]	83.39	73.88
0.5	(32, 96]	90.79	86.22
0.5	(96, 512]	90.29	71.90
0.5	(0, 512]	87.45	79.65

**Table 3:** Comparison of different detection frameworks.

Frameworks	*Precision* (%)	*Recall* (%)	*F*1-Score (%)	*mAP* (%)
Faster RCNN [2]	74.32	55.08	63.26	74.02
YOLOv5 [41]	78.80	75.00	76.85	81.70
RetinaNet [5]	78.37	70.56	78.37	79.39
RetinaNet-NeXt (Ours)	87.45	79.65	83.37	86.71

**Table 4:** Comparison of different models.

Backbone	*Precision* (%)	*Recall* (%)	*mAP* (%)
ResNet50	78.37	70.56	79.39
ResNet101	89.95	88.22	92.02
ResNet152	90.54	89.29	92.80
ResNeXt50	87.45	79.65	86.71

**Table 5:** Comparison of different models under the effect of anchor.

Backbone	*Precision* (%)	Anchor			
	*Recall* (%)	(0, 32]	(32, 96]	(96, 512]	(0, 512]
ResNet50	*Precision* (%)	68.73	85.85	88.94	78.37
	*Recall* (%)	60.22	79.83	75.81	70.56
ResNet101	*Precision* (%)	86.32	93.37	90.49	89.95
	*Recall* (%)	85.14	92.58	77.62	88.22
ResNet152	*Precision* (%)	86.85	94.09	90.58	90.54
	*Recall* (%)	86.53	93.50	77.53	89.29
ResNeXt50	*Precision* (%)	83.39	90.79	90.29	87.45
	*Recall* (%)	73.88	86.22	71.64	79.63

## Data Availability

All results and data obtained can be found in open access publications.
